# Supplementary material for: Bleaching‐resistant, Near‐continuous Single‐molecule Fluorescence and FRET Based on Fluorogenic and Transient DNA Binding
Source: Chemphyschem. 2023 Apr 12;24(12):e202300175. doi: 10.1002/cphc.202300175 (PMC10946581; doi:10.1002/cphc.202300175)
Supplement: Supplementary file 1 — Supporting Information [file CPHC-24-0-s001.pdf]

ChemPhysChem

Supporting Information

Bleaching-resistant, Near-continuous Single-molecule Fluorescence and FRET Based on Fluorogenic and Transient DNA Binding

Mirjam Kümmerlin, Abhishek Mazumder,* and Achillefs N. Kapanidis*

Bi-exponential fitting of binding kinetics.

To give readers straightforward numbers for the hybridisation kinetics in the main text, we have calculated mean values of t_{off} and t_{on}, and also the inverse values as k_{on} and k_{off}, respectively. From previous studies, we know, however, that the hybridisation kinetics are indeed better described by a bi-exponential decay with two independent decay constants. To further characterise the binding behaviour, we thus fitted a bi-exponential distribution to the dwell times (extracted as described in the main experimental section) of both r-labels using a maximum-likelihood-estimator in Matlab (MEMLET[1]). The fitted function takes into account the minimum measurable dwell time (one frame) and the discrete nature of the observable values. Confidence intervals for fitted decay times were determined via bootstrapping (Table S2). The results show that the green label at 100 nM has on-rates of $k_{on,1}$ =0.31 s^{-1} and $k_{on,2}$ =0.031 s^{-1} with 75.8% of events following $k_{on,1}$. The off-rate constant are $k_{off,1}$ =0.48 s^{-1} and $k_{off,2}$ =0.038 s^{-1} with 34.4% of all events following $k_{off,1}$ (see Figure S1A, C). For the red r-label, on-rates of $k_{on,1}$ =3.50 s^{-1} and $k_{on,1}$ =0.12 s^{-1} with 90.5% described by $k_{on,1}$ and off-rates of $k_{off,1}$ =0.90 s^{-1} (78.4%) and $k_{off,1}$ =0.031 s^{-1} were observed at 20 nM (Figure S1B, D).

Characterising self-quenching R-label strands. We additionally tested a construct terminally labelled with two ATTO655, two ATTO647N and Dabcyl, and ATTO647N and BHQ3. All constructs were 11-nt long, except the ATTO655 labelled probe, which had a length of 8 nt.

To characterise the level of quenching, we performed ensemble measurements, assessing the absorption spectra and the fluorescence of the quenched r-labels in absence and presence of up to 100-fold excess of complementary DNA.

The fluorescence spectra were measured at a scanning spectrofluorometer (PTI) using 1-s integration time per 1-nm-wavelength intervals using 100 µL r-labels in buffer (50 mM HEPES, pH 7.4; 200 mM $MgCl_2$, 10 mM NaCl, 0.1 % BSA, the same buffer as used for single molecule measurements) to a final concentration of 100 nM. Complementary DNA was added stepwise to achieve different concentrations until saturation of the signal was observed (0.1-10 µM). Samples were excited at 520 nm (containing Cy3B), 620 nm (containing ATTO647N) or 640 nm (containing ATTO655).

The emission spectra (Figure S2) were recorded in presence of 0 to 10 µM complementary DNA (complementary DNA was added until the fluorescence signal saturated). Upon hybridisation, the increased stiffness of dsDNA forces the dye-dye interactions apart and thus should de-quench the probes. All red r-labels (Figure S2A-D) show an increase in fluorescence

upon addition of an increasing concentration of complementary DNA, most prominent the 2xATTO647N and the ATTO647N-BHQ3 probes (4-fold and 6-fold increase, respectively). The level of fluorescence in the ATTO647N-BHQ3 probe is, however, still lower than the fully quenched 2xATTO647N. We reasoned that there is significant FRET from ATTO647N to the BHQ3 even in the hybridised state, which prevents fluorescence emission and thus renders the probe unsuitable for our purpose. For further single-molecule experiments, we selected the 2xATTO647N r-label as a red label, since it proved to be the most suitable out of the tested selection.

For the green r-label, we observe a 16-fold increase in fluorescence intensity under the same conditions (Figure S2E), which suggests that indeed increasing the distance between dark quencher and fluorophore in the de-quenched state (compared to the ATTO647N-BHQ3 pair) is allowing for strong emission once hybridised with complementary DNA.

REFERENCES

[1] M. S. Woody, J. H. Lewis, M. J. Greenberg, Y. E. Goldman, E. M. Ostap, *Biophys J* **2016**, *111*, 273–282.

Table S1: Sequences of DNA strands. The HJ is formed by nucleotides in capitals, other nucleotides are involved in r-label binding.

**Strand name**	**Sequence**	**Modification**
HJ-B	5′-CCCTAGCAAGCCGCTGCTACGG	--
HJ-H	5′-CCGTAGCAGCGAGAGCGGTGGG	5'-biotin
HJ-R	5′-CCCACCGCTCTTCTCAACTGGG	5'-ATTO647N
HJ-X	5′-CCCAGTTGAGAGCTTGATAGGG	5'-Cy3B
HJ-Rl	5′-aa aaa ggg aaa-<br>CCCACCGCTCTTCTCAACTGGG	--
HJ-Xl	5′- ttc aac att tct tct<br>CCCAGTTGAGAGCTTGATAGGG	--
rl-2xATTO647N	5'-ttt ccc ttt tt	5' and 3' ATTO647N
rl-ATTO647N-Dabcyl	5'-ttt ccc ttt tt	5' ATTO647N, 3'Dabcyl
rl-ATTO647N-BHQ3	5'-ttt ccc ttt tt	5' ATTO647N, 3' BHQ3
comprl	5'-aa aaa ggg aaa	--
gl-Cy3B-BHQ2	5'-aga agt aat gtg gaa	5' Cy3B, 3' BHQ2
compgl	5′- ttc aac att tct tct	--
8mer-2xATTO655	5'-tcc acc gt	5' and 3' ATTO655
Comp-8mer	5'-ac ggt gga	--

Table S2: Parameter mean and 95% CI from bootstrapping (200 iterations) the binding kinetics of the r-labels.

label	state	parameter	mean	lower 95% CI	upper 95% CI
green	off	A	0.757	0.752	0.761
		t1 (in s)	3.189	3.151	3.227
		t2 (in s)	31.080	30.616	31.544
	on	A	0.341	0.337	0.345
		t1 (in s)	1.491	1.455	1.528
		t2 (in s)	24.907	24.706	25.108
red	off	A	0.905	0.905	0.906
		t1 (in s)	0.285	0.284	0.285
		t2 (in s)	8.051	8.005	8.098
	on	A	0.783	0.783	0.784
		t1 (in s)	1.105	1.101	1.108
		t2 (in s)	31.708	31.571	31.845

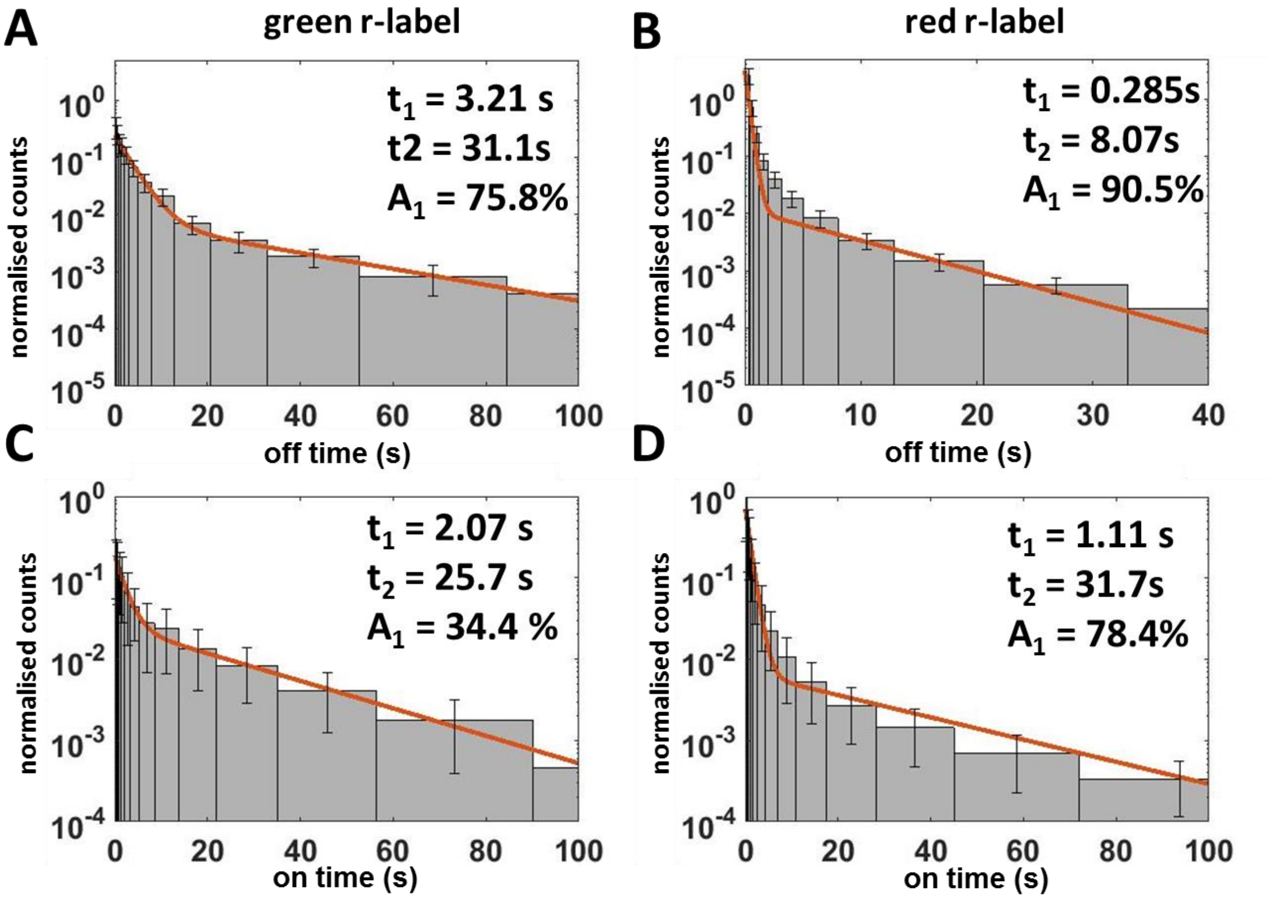

Figure S1: Characterisation of the binding kinetics for the r-labels. The data was fitted to a bi-exponential distribution using a MLE algorithm in Matlab (MEMLET[1], red graph). For display purposes, the data were binned with increasing bin size, counts were normalised by the bin width. Error bars on bins are derived from bootstrapping (200 iterations).

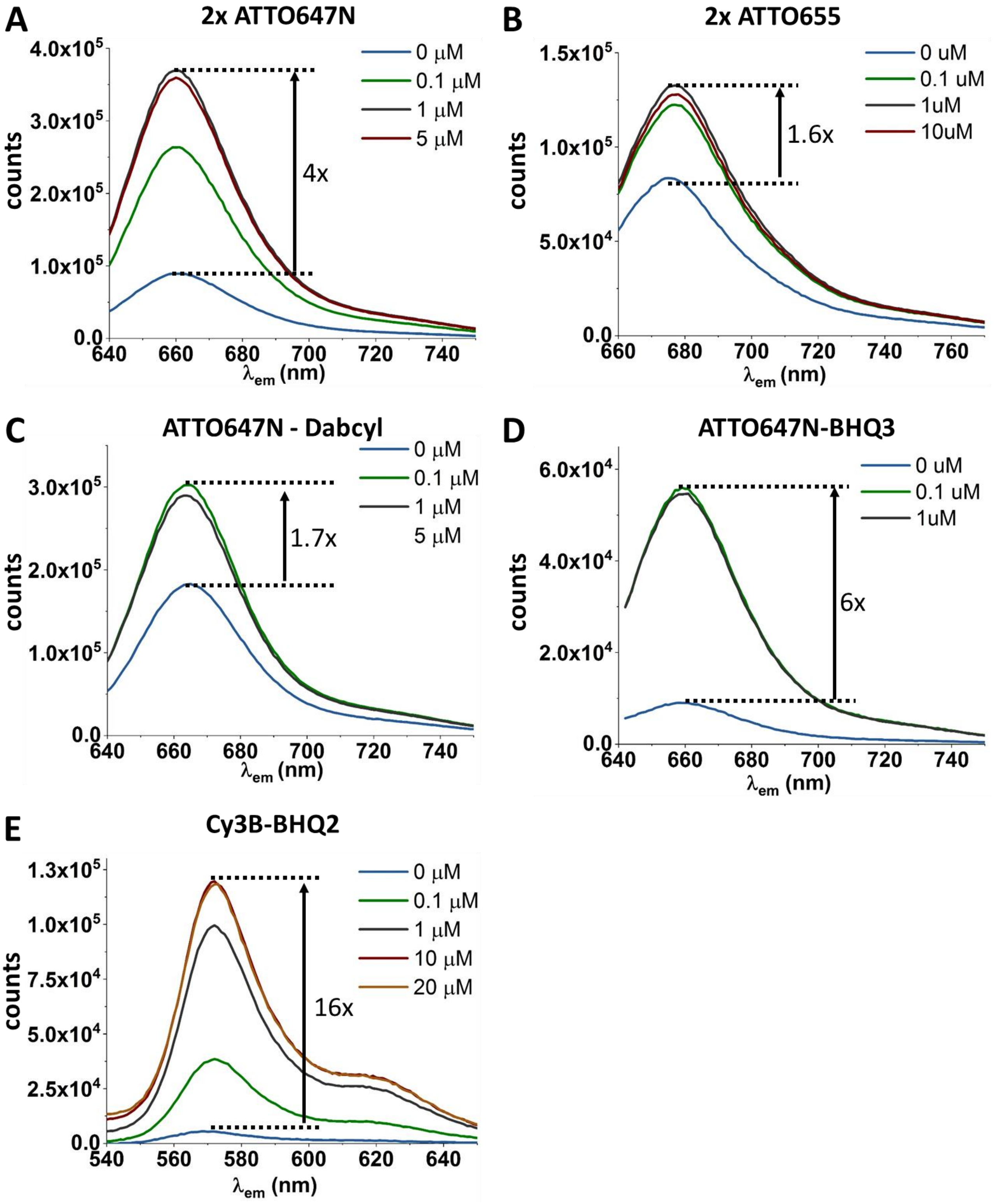

Figure S2: Characterisation of quenching efficiency in double-labelled r-label strands. Fluorescence spectra of probes terminally labelled with two ATTO647N (**A**), two ATTO655 (**B**), ATTO647N and Dabcyl (**C**), ATTO647N and BHQ3 (**D**), or Cy3B and BHQ2 (**E**) at various concentrations of complementary ssDNA.

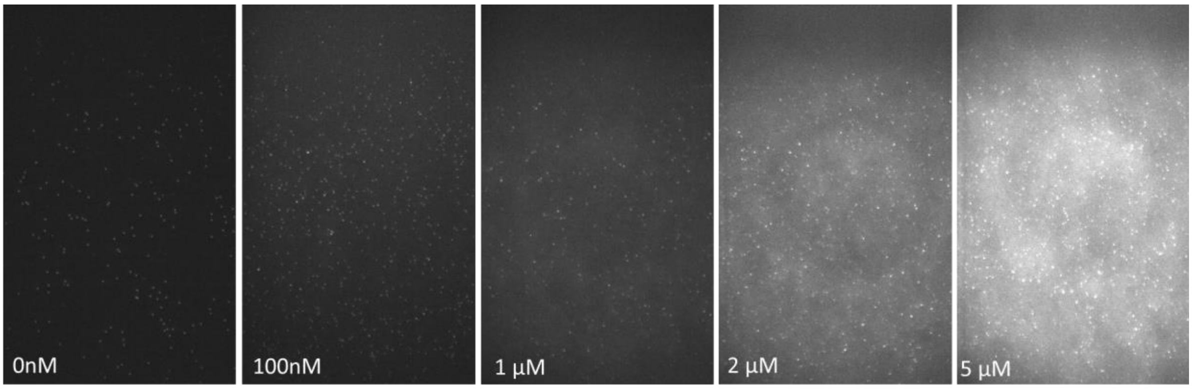

Figure S3: Individual frames imaged at various concentrations of green r-labels (0-5µM) in the imaging buffer. At 0 nM, the observed spots come from immobilised Cy3B. The subsequence images show spots of green r-labels at various concentrations binding to complementary, immobilised docking strands. Through the fluorogenic nature of the r-lables, individual targets can still clearly be identified at 5µM of r-lables in the imaging buffer.

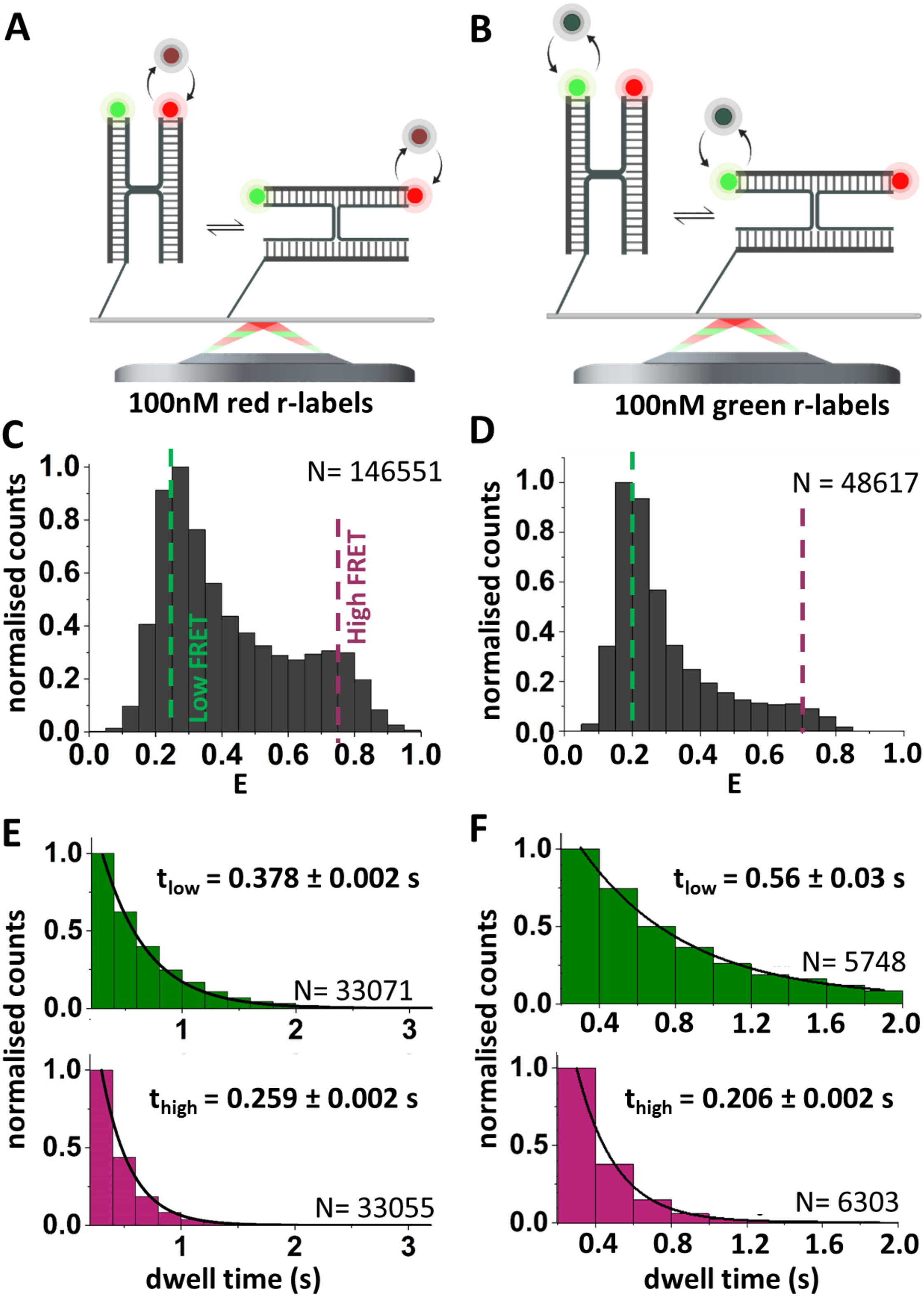

Figure S4: Conformational dynamics in the HJ observed with single r-labels. FRET distributions (**C-D**) and dwell time histograms (**E-F**) for the HJ with only (**A, C, E**) or green (**B, D, F**) r-label, as indicated by the schematics in **A** and **B**, respectively. Data from one representative experiment of 179 (red r-label) and 72 molecules (green r-label).

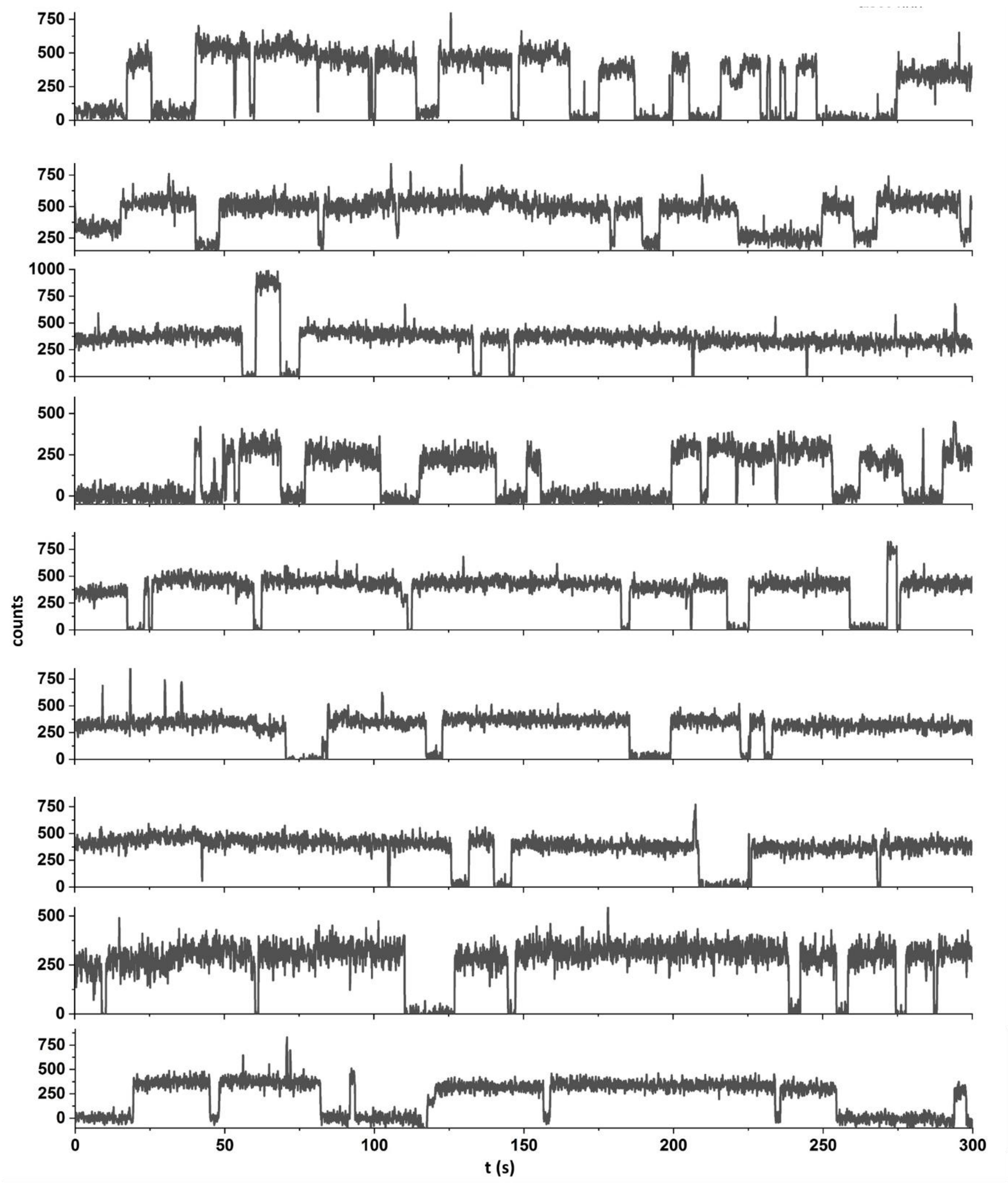

Figure S5: Additional traces of the green r-label binding at 100 nM. Repeated binding can be observed by the signal rising to ≈450 counts. Occasionally, the signal reaches higher counts (≈ 800 counts), which indicates intervals of Cy3B fluorescence without functional BHQ2.

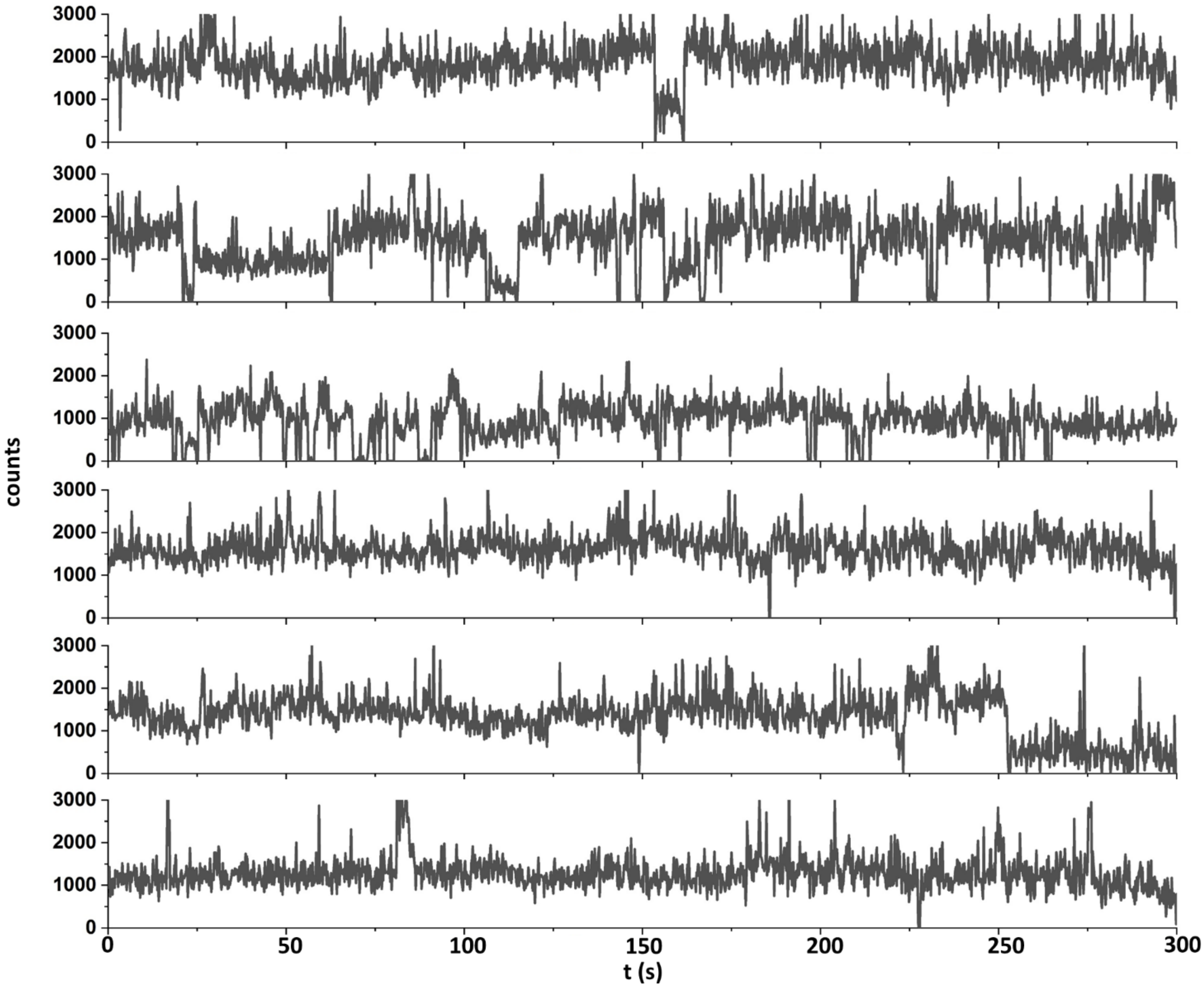

Figure S6: Additional traces of the red r-label binding (100 nM). Repeated binding can be observed by the signal rising to ≈1000 - 1500 counts. Occasionally, the signal shows lower counts (≈ 750 counts), which indicates intervals of only one functional ATTO647N dye emitting.

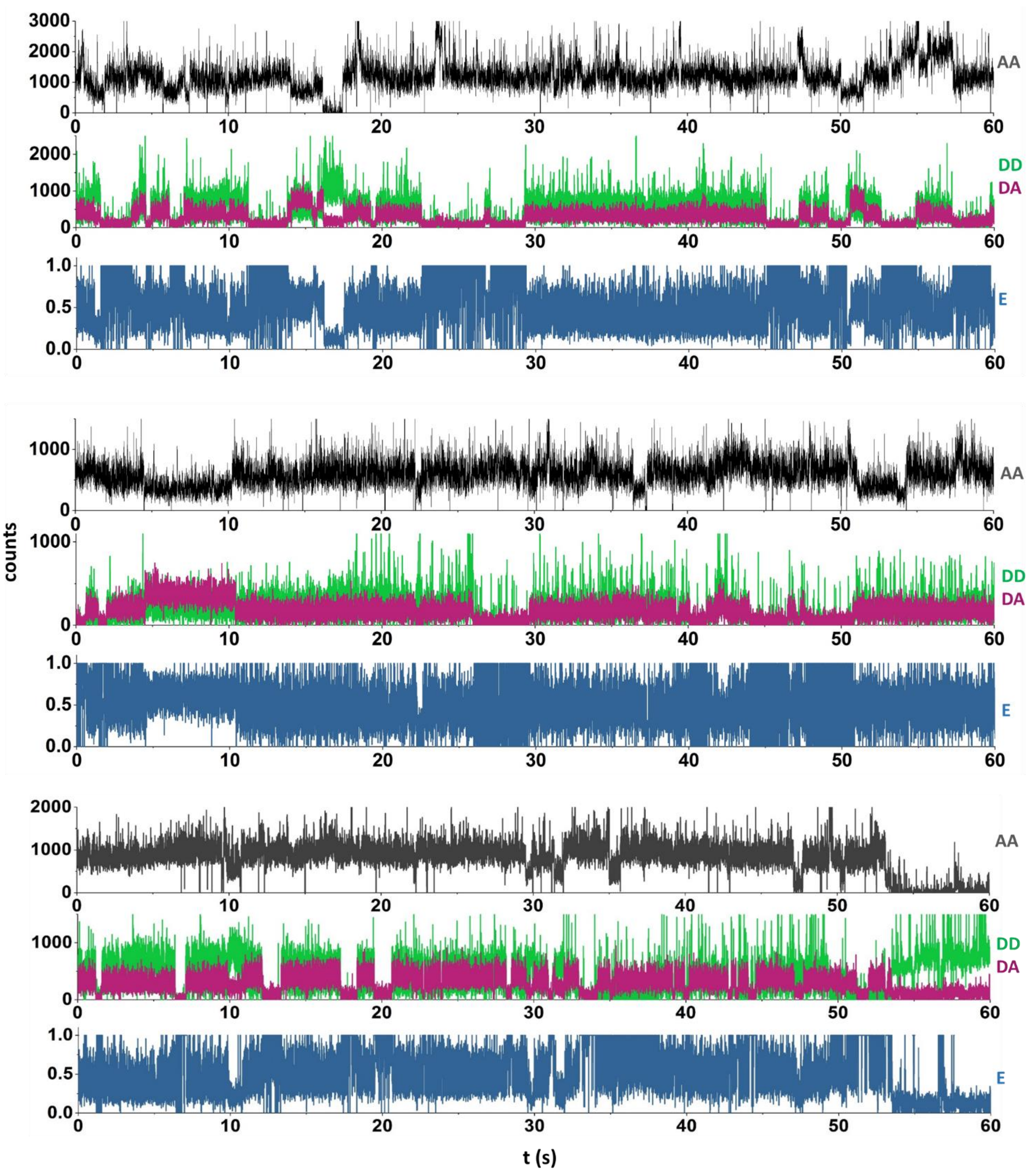

Figure S7: Additional traces of the green (300 nM) and red r-label (100 nM) binding in the FRET regime on the HJ with AA (top, grey), DD, and DA channel (middle, green and magenta, respectively) and calculated FRET efficiency (E, bottom, blue). The anti-correlated fluctuations in the DD and DA channels (middle) and the fluctuating E trace (bottom) indicate a dynamic interchange between a high-FRET state (E ≈ 0.75) and a low-FRET state (E ≈ 0.25).
